# Food insecurity, nutritional status and socioeconomic factors in the transgender population: a cross-sectional study in the Metropolitan Region of Baixada Santista, Brazil, 2023

**DOI:** 10.1590/S2237-96222024v33e2024371.especial.en

**Published:** 2024-11-04

**Authors:** Ísis Gois, Magnus Régios Dias da Silva, Barbara Iansã de Lima Barroso, Carla Gianna Luppi, Denise Leite Vieira, Katia Cristina Bassichetto

**Affiliations:** 1 Universidade Federal de São Paulo, Núcleo de Estudos, Pesquisa, Extensão e Assistência à Pessoa Trans Professor Roberto Farina, São Paulo, SP, Brazil; 2 Universidade Federal de São Paulo, Departamento de Medicina Preventiva, São Paulo, SP, Brazil; 3 Faculdade de Ciências Médicas da Santa Casa de São Paulo, São Paulo, SP, Brazil

**Keywords:** Personas Transgénero, Inseguridad Alimentaria, Estado Nutricional, Factores Socioeconómicos, Transgender People, Food Insecurity, Nutritional Status, Socioeconomic Factors

## Abstract

**Objective:**

To describe the distribution of nutritional status and food insecurity among the adult transgender population in the Baixada Santista region of the state of São Paulo and to identify associated factors.

**Methods:**

This was a cross-sectional study using data from the research project entitled Mapping the Transgender Population in Baixada Santista of the state of São Paulo, conducted through a structured questionnaire administered between August and December 2023. The outcomes were nutritional status and food and nutrition insecurity (FNI). The association analysis was performed using Fisher’s exact test.

**Results:**

A total of 237 people took part in the study. High prevalence of FNI was associated with an income of less than 2 minimum wages (p < 0.001), difficulty finding a job (p < 0.001) and lack of family support related to gender (p = 0.001). Difficulty reading/writing (p = 0.025) and proximity to an open-air market (p = 0.033) were negatively or positively associated with adequate nutritional status, respectively.

**Conclusion:**

The high prevalence of FNI among the most vulnerable population and the adequate nutritional status associated with proximity to open-air markets indicate the need for policies aimed at reducing inequities and expand access to adequate food.

## Study contributions

**Main results:**

The high prevalence of FNI was associated with low income, difficulty finding a job and lack of family support. Adequate nutritional status among the transgender population analyzed was associated with proximity to open-air markets.

**Implications for services:**

Service teams should incorporate the assessment of nutritional status and food and nutrition security into their routine practices in order to provide adequate service that addresses the demands and social situation of the transgender people they serve.

**Perspectives:**

There is a need for public policies aimed at reducing socioeconomic disparities exacerbated by discrimination against transgender people and facilitating access to establishments that offer diverse and quality food.

## RESUMEN

**Objetivo:**

Describir la distribución del estado nutricional y de inseguridad alimentaria de la población adulta trans en la región de Baixada Santista/Estado de San Pablo, además, identificar factores relacionados.

**Métodos:**

Datos del estudio transversal Mapeo de la Población Trans en Baixada Santista de San Pablo, realizado mediante cuestionario estructurado entre agosto-diciembre de 2023. Los resultados fueron el estado nutricional y la inseguridad alimentaria y nutricional (IAN). Análisis de asociación mediante la prueba exacta de Fisher.

**Resultados:**

Participaron del estudio 237 personas. La alta prevalencia de IAN se asoció con ingresos inferiores a 2 salarios mínimos (p < 0,001), dificultad para encontrar trabajo (p < 0,001) y falta de apoyo familiar relacionado con el género (p = 0,001). La dificultad para leer/escribir (p = 0,025) y acceso a un mercado callejero (p = 0,033) se asociaron negativa o positivamente con un estado nutricional adecuado, respectivamente.

**Conclusión:**

Alta prevalencia de NIA entre la población más vulnerable y adecuación del estado nutricional asociado al acceso a mercados callejeros cercanos indican la necesidad de políticas que decrezcan las inequidades y extiendan el acceso a una alimentación adecuada.

## INTRODUCTION

Food and nutrition security is defined as “regular and permanent access to quality food, in sufficient quantity, without compromising access to other essential needs”.^1^ Based on this concept, food and nutrition insecurity (FNI) is understood as a social problem that affects comprehensive health, due to the psycho-emotional and biological consequences associated with nutritional deficiencies, overweight and obesity.^2^.^3^

Several factors can contribute to this situation. With regard to agricultural production and the fight against hunger, Brazil persists in following outdated directives of a predatory economy, expanding subsidized financing for the agribusiness sector and directing food production for export.^4,5^ These decisions only benefit large corporations and their respective intermediaries, without taking into account the need to produce quality food for the population’s food security. Thus, reducing inequalities requires reorienting the economy to meet the needs of the population, primarily through policies to combat hunger.^4^

Furthermore, the characteristics of the food environment can interfere with physical access to healthy foods and accentuate health inequalities, leading to an increase in the consumption of unhealthy foods, due to the greater availability of establishments selling ultra-processed foods compared to those selling healthier options.^5^

Regarding the COVID-19 pandemic, it can be stated that, even before this exacerbating factor, the transgender population was already considered a vulnerable group for FNI,^5,6,7^ but this pandemic has further affected this reality.^7,8,9^ One of the first studies on FNI among the Brazilian transgender population found a prevalence of 68.8% in the sample. In addition, transgender people have other nutritional and social aspects that are shaped by gender experiences and there are gaps in the scientific literature on this topic, especially in Global South countries.^7,6^

Thus, the objective of this study was to describe the distribution of nutritional status and food insecurity among the adult transgender population (aged 18 years and old) in the Baixada Santista region, state of São Paulo, and to identify associated factors.

## METHODS

### Study design and participants

This study used data from the research project entitled Mapping the Transgender Population of the State of São Paulo: Metropolitan Region of Baixada Santista (MTP), a cross-sectional observational study, employing both a quantitative and qualitative approach that mapped the transgender population aged 18 years or old, living, working or studying in one of the nine municipalities that comprise this region of the state of São Paulo. The MTP investigated the socioeconomic and demographic characteristics of this population, including living conditions, work, health and related aspects.

### Sample

The value of 1.88% was applied to the total adult population of the municipalities in Baixada Santista, the only national estimate available of the magnitude of transgender and non-binary people, obtained from a Brazilian estimate.^10^ Based on 19,965 people with an expected frequency of 50%, and a design effect of 1, a sample of 377 people was calculated, proportionally distributed across the municipalities.

### Recruitment and data collection

An online registration of interest in taking part in the research was used as a recruitment strategy. The link and QR code for accessing the registration were widely disseminated in the Baixada Santista region and on social media (Instagram and Meta® WhatsApp groups). Transgender, transvestites and non-binary people who completed this registration and responded positively to the question *Can we contact you?* by providing contact information were invited to participate in this study.

In order to enable contact with those who had agreed to participate in the study, an Excel spreadsheet was generated from the database on the REDCap (Research Electronic Data Capture) platform, which was updated weekly and made available to the field supervisor and a trained interviewer for scheduling. These contacts were made via WhatsApp (Meta®), email or telephone, and for those who agreed to take part, an interview was scheduled, either face-to-face or remotely, using the Zoom ® platform, depending on their preference.

In addition to registration, active searches were conducted at primary health care centers specialized in transgender health care, as well as locations serving as reference points for welcoming this population in the study region.

Data collection involved administering a structured questionnaire on the REDCap platform. The complete questionnaire had 11 thematic blocks (Box 1). For this study, we used the variables from blocks 1, 2 and 4.

**Box 1:** Thematic blocks of the questionnaire used in the research entitled Mapping of the Transgender Population of the State of São Paulo: Metropolitan Region of Baixada Santista, Brazil, August to December 2023

Blocks
Socioeconomic and demographic characteristics
Access to health services and support network
Diseases, oral health problems and disabilities
Nutritional status and access to food
Body Modifications
Sexual behavior
Sexual, reproductive health and prevention strategies
Sexually transmitted infections (STIs)
Quality of life, physical and psychosocial activity
Use of alcohol, tobacco and other substances
Intersectional stigma

The data collection period for this study, planned for October and November 2023, was extended by one month to approximate the initially calculated sample size. However, the sample was not achieved in all municipalities, and, due to the exhaustion of recruitment strategies, it was decided to conclude the fieldwork.

### Variables

The primary outcomes of this study were nutritional status (NS) and FNI. In order to analyze socioeconomic and demographic characteristics, the following variables were selected: age (18 to 24, 25 to 30 and over 30 years old); gender identities collected as trans woman, transvestite, trans man, transmasculine individual, non-binary person, agender and pangender and categorized into “transfeminine” (trans women and transvestites), “ transmasculine” (trans man and transmasculine individual) and “non-binary” (non-binary person, agender and pangender); self-declared race/skin color; schooling (incomplete and complete elementary education, incomplete and complete high school, incomplete and complete higher education, and postgraduate degree); income in the last month (no income, up to one minimum wage and above one minimum wage); difficulty finding a job (yes or no); source of income (internship/scholarship, formal work (Consolidation of Labor Laws – Consolidação das Leis do Trabalho - CLT, legal entity – LE, public servant, contract, informal work and benefits (pension, Bolsa Família, sickness benefit and social security retirement); and existence of family support, considering the respondent as a trans person (family does not know about transgenderism, fully supportive, partially supportive, unsupportive, indifferent, and has no contact with family/completely disapproves).

Nutritional status was analyzed using the body mass index (BMI) [weight/height^2^ (kg/m^2^)], according to the World Health Organization (WHO) classification for the adults, which categorizes the population into underweight grade III (BMI < 16), underweight grade II (BMI 16 to 16.99), underweight grade I (BMI 17 to 18.49), eutrophy (ideal weight for height) (18, 50 to 24.99), overweight (25 to 29.99), grade I obesity (30.00 to 34.99), grade II obesity (35.00 to 39.99) and grade III obesity (over 40) . These categories were regrouped into underweight (BMI < 18.50 kg/m^2^ ), eutrophy (18.50 to 24.99 kg/m^2^), overweight (24.99 to 30.00 kg/m^2^) and obesity (BMI above 30.00 kg/m^2^);^11^ the BMI classification for individuals aged 60 years and older is categorized as underweight (BMI < 22.0 kg/m^2^ ), eutrophy (22.0 to 27.0 kg/m^2^ ) and overweight/obesity (BMI above 27 kg/m2);^11^ degree of FNI was calculated according to the Brazilian Food Insecurity Scale - 2014 (eight questions) and classified as mild (1-3 points), moderate (4-5 points) and severe (6-8 points);^4^.^5^ and, to assess the quality of access, they were asked about the types of establishments near their residence that sell fruits and vegetables.^12^

### Statistical analysis

Results were presented descriptively, including frequency, percentage, mean and standard deviation. Fisher’s exact test was used to analyze the factors associated with the outcomes, considering a p-value < 0.05 for statistical significance. Data were analyzed using the Jamovi 2.3.21 statistical *software.* ^14^

### Ethical aspects

The MTP research project was submitted to and approved by the Research Ethics Committees of the Centro de Referência e Treinamento em IST/Aids de São Paulo (CRT) and the Universidade Federal de São Paulo, under Certificate of Submission for Ethical Appraisal No. 64010722.8. 0000.5375.

## RESULTS

The sample consisted of 237 people, representing 62.9% of the calculated sample, from the nine municipalities of the Metropolitan Region of Baixada Santista. The most represented municipalities were Santos (27.8%), São Vicente (23.6%) , Guarujá (23.2%), Praia Grande (8.9%) and Itanhaém (8.9%). Regarding gender identity, 42.6% of people identified as transfeminine; 36.3%, as transmasculine; and 21.1%, as non-binary. The median age was 27 years (minimum 18 and maximum 68 years old), and 67.5% were under 30 years old.

Table 1 shows selected socioeconomic and demographic characteristics. Regarding the level of education, 80.5% had completed at least high school. However, 5.5% of the sample reported difficulty reading and writing. With regard to income and the labor market, 65.8% stated that they had difficulty finding a job, 63.7% reported having no income or an income of up to one minimum wage in the 30 days prior to the interview, and among those who had a source of income, 46.3% reported formal employment.

**Table 1:** Socioeconomic, demographic and nutritional characteristics (n and %) of transgender people taking part in the Mapping of the Transgender Population of the State of São Paulo: Metropolitan Region of Baixada Santista, Brazil, August to December 2023

Variable	Frequency (n)	Percentage (%)
Gender identity (n = 237)		
Transfeminine	101	42.6
Transmasculine	86	36.3
Non-binary	50	21.1
Age group (n = 221)		
18 to 24 years old	83	37.6
25 to 30 years	66	29.9
Over 30 years old	72	32.6
Race/skin color (n = 237)		
White	121	51.1
Black	107	45.1
Other	9	7.8
Schooling (n = 237)		
Incomplete elementary education	16	6.8
Complete elementary education	8	3.4
Incomplete high school	22	9.3
Complete high school	92	38.8
Incomplete higher education	61	25.7
Complete higher education	24	10.1
Postgraduate degree	14	5.9
Income in the last month (n = 236)		
No income	77	32.5
Up to 1 minimum wage	74	31.2
Above 1 minimum wage	85	35.9
Source of income (n = 160)		
Scholarship/internship	6	3.7
Social and welfare benefits	18	11.3
Informal work	62	38.7
Formal work	74	46.3
Difficulty finding a job (n = 237)		
Yes	156	65.8
No	73	30.8
Don’t know/did not answer	8	3.4
Family support (n = 235)		
Fully supportive	82	34.9
Partially supportive	76	32.3
Do not know	12	5.1
Indifferent	31	13.2
Unsupportive	19	8.1
Completely disapproves	15	6.4
Nutritional status (n = 236)		
Underweight	6	2.5
Eutrophy	110	46.6
Overweight	76	32.2
Obesity	44	18.6
Food insecurity (n = 237)		
Yes	150	63.3
No	87	36.7
Degree of food insecurity (n = 150)		
Mild	70	46.7
Moderate	32	21.3
Severe	48	32.0
Places that sell fruits and vegetables near the residence (n = 237)		
Supermarket/hypermarket	126	53.2
Neighborhood market	161	67.9
Produce market/greengrocer	107	45.1
Open-air market	155	65.4
Street vendors	51	21.5
Municipal market	10	4.2
There are no such establishments	7	3
Do you buy fruits and vegetables at nearby locations (n = 237)		
Yes	202	85.2
No	29	12.3
I do not buy fruits and vegetables	6	2.5

Full (34.9%) and partial (32.3%) family support for gender identity was indicated by 67.2% of participants. Approximately 5% reported that their family was unaware of their gender identity and 27.7% reported some form of lack of family support. 

Participants had an average BMI of 25.7 ± 5.57 kg/m^2^ and 50.8% were overweight or obese. FNI was present in nearly 65% of the sample, with 53.3% of those categorized as having moderate and severe FNI (Table 1).

Regarding access to places selling fruit and vegetables near the participant’s residence, the most frequently mentioned were neighborhood markets, open-air markets, hypermarkets and large supermarket chains and greengrocers, respectively. A total of 14.8% of the participants reported either not buying fruits and vegetables, or not buying them near their residences (Table 1).

**Figure 1:**
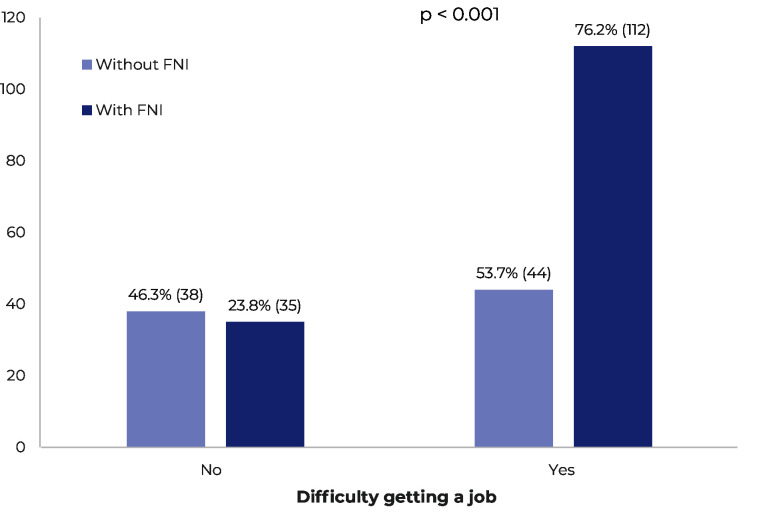
Food insecurity among the transgender population according to difficulty finding a job, bases on data from the Mapping of Transgender Population of the State of São Paulo: Baixada Santista Region, Brazil, from August to December 2023, with values expressed in frequency (n) and percentage (%)

**Figure 2:**
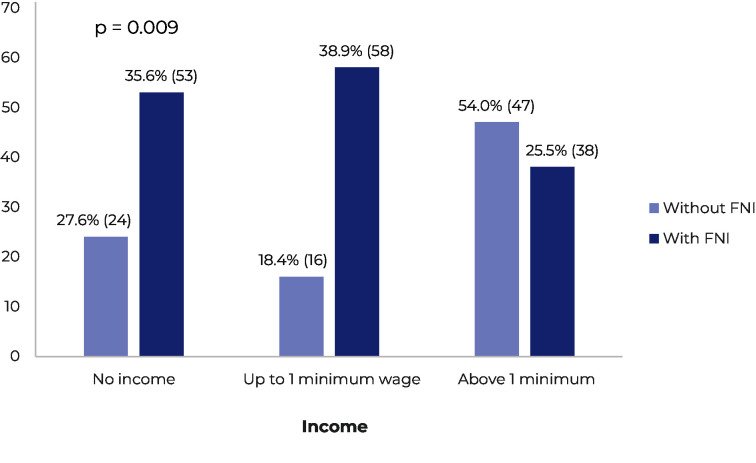
Food insecurity among the trans population according to income levels in the last 30 days, based on data from the Mapping of the Transgender Population of the State of São Paulo: Baixada Santista Region, Brazil, from August to December 2023, with values expressed in frequency (n) and percentage (%)

Taking into consideration the bivariate analysis, factors associated with the presence of FNI included difficulty finding a job (p < 0.001) (Figure 1) and having no income in the month prior to the interview (p = 0.009) (Figure 2). Furthermore, participants who reported earning less than 2 minimum wages (81%) were more likely to experience some degree of FNI (p < 0.001). It is worth highlighting that lack of income was associated with the age group of 18 to 24 years (p = 0.001).

In addition to the aspect of income and employability, a positive association between lack of family support and the presence of FNI was observed. Participants who reported partial or total family support were less likely to experience FNI (p = 0.006); on the other hand, those who reported indifference, lack of support or disapproval from the family due to being trans, were more likely to have moderate and severe FNI (p = 0.001). Another aspect related to these degrees of FNI was the report of not buying fruits and vegetables near the residence or not routinely purchasing fruit and vegetables (p = 0.050).

**Figure 3:**
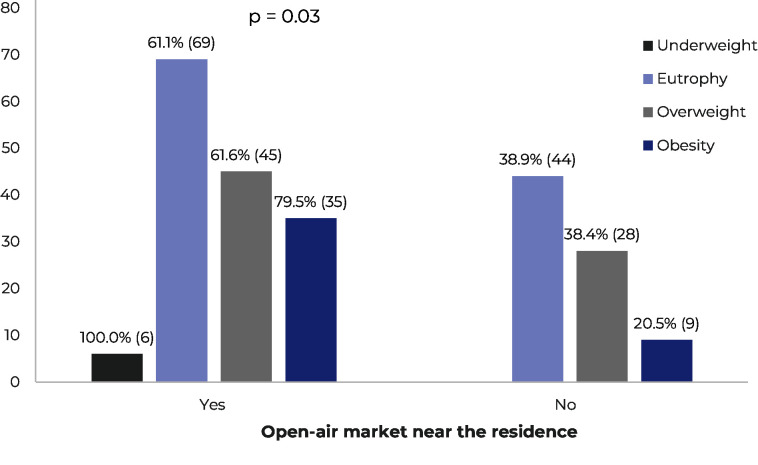
Classification of nutritional status according to the presence of an open-air market near the residence, based on data from the Mapping of the Transgender Population of the State of São Paulo: Baixada Santista Region, Brazil, from August to December 2023, with values expressed in frequency (n) and percentage (%)

Nutritional status was associated with certain socioeconomic factors. Difficulty reading and writing was associated with being overweight and negatively associated with eutrophy (p = 0.025). Moreover, people with access to establishments that sell fruit and vegetables, such as the presence of a street market near their residence, were more likely to be classified as having adequate nutritional status compared to those classified as overweight and obese (p = 0.033) (Figure 3).

In this sample, race/skin color and gender identity variables were not associated with any socioeconomic and food insecurity variables.

## DISCUSSION

The study identified high prevalence of FNI in the study population, which was associated with income, difficulty finding a job and lack of family support related to gender, as well as aspects of access to the purchase of vegetables and fruits.

In Brazil, according to the Food and Agriculture Organization of the United Nations (FAO) report, approximately 34% of the population experiences FNI.^15^ However, data regarding FNI among the transgender population in the country is twice as high.^8^ This study, conducted during the COVID-19 pandemic, found prevalence of FNI in a sample of 109 Brazilian transgender people from all regions of the country similar to that of this study (68.8% versus 63.3%). The authors emphasize that access to adequate food was already difficult for this population, even before the pandemic, and the main associations found for this prevalence were due to income and unemployment,^8^ similar to the present study.

In order to discuss high social vulnerability and FNI in the transgender population, it is necessary to take into account discrimination based on gender identity,^8^ as well as socioeconomic disparities between transgender people, which may be associated with passability related to the duration of transition, social acceptance and family support.^8^ The high proportion of people experiencing FNI in this study, associated with a lack of family support, high rates of difficulty finding a job and, consequently, low income, are probably related to discrimination based on gender identity and structural transphobia.^7^.^8^

With regard to nutritional status, a systematic review showed that there is higher prevalence of overweight and obesity among the transgender population, especially after the use of sexual steroids for phenotypic and body changes.^7^ In this study, we did not assess the use of hormones. However, the prevalence and distribution of nutritional status, according to BMI classification categories, were similar to those of the general adult population in the city of São Paulo, despite the fact that the considered database being from 2015, derived from the ISA Capital Population-Based Health Survey.^16^

Furthermore, the eutrophic nutritional status of the sample was associated with the presence of open-air markets near the residence. A Brazilian study showed that an environment with favorable access to a greater variety of healthy foods, such as fruits and vegetables, can promote the prevalence of lower overweight and obesity.^17^

Regarding the limitations of the study, the weight and height data used to calculate BMI were self-reported. In the sample, applying the same percentage across all municipalities may not correspond to the actual distribution of this population in the territory. In addition, as the sample came from just one metropolitan region in the state of São Paulo and reached only 62.9% of the calculated sample, the conclusion of this study may not be representative of the entire adult transgender population.

There is a scarcity of research on general nutritional aspects in the transgender population, especially in the least developed countries located in the Global South.^6^ Furthermore, national surveys conducted by the Brazilian Institute of Geography and Statistics (*Instituto Brasileiro de Geografia e Estatística* - IBGE) do not include transgender categories. Thus, this study contributes to the literature by filling a gap in data on nutritional status and FNI in this population, while also providing a basis for comparison with other studies, as we use validated measures. Therefore, the data provided by this study can contribute to informed discussions that guide the need for policies aimed at reducing the high prevalence of FNI observed in this population.

Thus, it is recommended that health service teams, especially nutrition and social care professionals, be vigilant in assessing the nutritional status and signs of FNI, in order to provide adequate care that meets the demands and social status of transgender people.^6^.^18^ The data from this study also corroborate the need for specific intersectoral public policies, aimed at reducing socioeconomic disparities exacerbated by discrimination,^9^ which contributed to a higher rate of FNI in the transgender population. In addition, it is necessary to develop policies aimed at facilitating access to open-air markets and other establishments that offer diverse and quality food.

These recommendations align with the proposals outlined at the 16^th^ National Health Conference regarding the training of health teams to care for LGBTI+ people and the implementation of public health policies targeting this population.^19^

The main findings of this study were the high prevalence of FNI among transgender people, in association with those who were more socially vulnerable and who reported a lack of family support due to their gender identity. On the other hand, adequate nutritional status was associated with the presence of establishments such as open-air markets near the residence of this population. These data underscore the need for public policies and actions aimed at reducing social inequities and expanding access to adequate food for the transgender population.
