# Supplementary material for: Food insecurity, nutritional status and socioeconomic factors in the transgender population: a cross-sectional study in the Metropolitan Region of Baixada Santista, Brazil, 2023
Source: Epidemiol Serv Saude. 2024 Nov 4;33(spe1):e2024371. doi: 10.1590/S2237-96222024v33e2024371.especial.en (PMC11534073; doi:10.1590/S2237-96222024v33e2024371.especial.en)
Supplement: Translation

# Translation: Insegurança alimentar, estado nutricional e fatores socioeconômicos na população trans: um estudo transversal na Região Metropolitana da Baixada Santista em 2023

**DOI:** 10.1590/S2237-96222024v33e2024371.especial.pt

**Authors:** Ísis Gois, Magnus Régios Dias da Silva, Barbara Iansã de Lima Barroso, Carla Gianna Luppi, Denise Leite Vieira, Katia Cristina Bassichetto

**Affiliations:** 1 Universidade Federal de São Paulo, Núcleo de Estudos, Pesquisa, Extensão e Assistência à Pessoa Trans Professor Roberto Farina, São Paulo, SP, Brasil; 2 Universidade Federal de São Paulo, Departamento de Medicina Preventiva, São Paulo, SP, Brasil; 3 Faculdade de Ciências Médicas da Santa Casa de São Paulo, São Paulo, SP, Brasil

**Keywords:** Pessoas Transgênero, Insegurança Alimentar, Estado Nutricional, Fatores Socioeconômicos

## Abstract

**Objetivo:**

Descrever a distribuição do estado nutricional e insegurança alimentar da população trans adulta da região da Baixada Santista, no estado de São Paulo, e identificar fatores associados.

**Métodos:**

Dados provenientes do estudo transversal *Mapeamento da População Trans* na Baixada Santista de São Paulo, conduzido com aplicação de questionário estruturado entre agosto e dezembro de 2023. Os desfechos foram estado nutricional e insegurança alimentar e nutricional (IAN). Foi realizada análise de associação pelo teste exato de Fisher.

**Resultados:**

Participaram do estudo 237 pessoas. Alta prevalência de IAN esteve associada à renda menor do que 2 salários mínimos (p < 0,001), dificuldade de encontrar emprego (p < 0,001) e falta de apoio familiar relacionada ao gênero (p = 0,001). Foram associados negativamente ou positivamente à adequação do estado nutricional dificuldade de ler/escrever (p = 0,025) e acesso próximo a feiras livres (p = 0,033), respectivamente.

**Conclusão:**

A alta prevalência de IAN entre a população mais vulnerabilizada e o estado nutricional adequado associado à presença de feiras livres próximas indicam necessidade de políticas para a redução de iniquidades e ampliação do acesso à alimentação adequada.

## Contribuições do estudo

**Principais resultados:**

A alta prevalência de IAN esteve associada à baixa renda, dificuldade de encontrar emprego e falta de apoio familiar. O estado nutricional adequado da população trans analisada esteve associado ao acesso próximo a feiras livres.

**Implicações para os serviços:**

As equipes de serviços devem inserir na rotina a avaliação do estado nutricional e segurança alimentar e nutricional para prover um atendimento adequado às demandas e à situação social das pessoas trans atendidas.

**Perspectivas:**

Há necessidade de políticas públicas direcionadas à redução das disparidades socioeconômicas, aprofundadas pela discriminação da transgeneridade, e para facilitação do acesso a estabelecimentos que oferecem variedade e qualidade alimentar.

## INTRODUÇÃO

A segurança alimentar e nutricional é definida como o “acesso regular e permanente a alimentos de qualidade, em quantidade suficiente, sem comprometer o acesso a outras necessidades essenciais”.^1^ A partir deste conceito, entende-se a insegurança alimentar e nutricional (IAN) como um problema de ordem social que afeta a saúde integral, em razão das consequências psicoemocionais e biológicas associadas a carências nutricionais, sobrepeso e obesidade.^2,3^

Vários fatores podem contribuir para essa situação. No que se refere à produção agrícola e ao combate à fome, o Brasil continua a manter velhas diretrizes da economia predatória, ampliando o financiamento subsidiado para o setor do agronegócio e direcionando a produção de alimentos para a exportação.^4,5^ Tais decisões beneficiam apenas grandes corporações e seus respectivos intermediários, sem levar em conta a necessidade de produzir alimentos de qualidade para a segurança alimentar da população. Portanto, a redução das desigualdades requer que a economia seja reorientada para atender às necessidades da população, passando, essencialmente, por políticas de combate à fome.^4^

Além disso, as características do ambiente alimentar podem interferir no acesso físico a alimentos saudáveis e acentuar as desigualdades em saúde, levando a um aumento do consumo de alimentos não saudáveis, em função da maior disponibilidade de estabelecimentos de comercialização de alimentos ultraprocessados em relação aos que oferecem opções mais saudáveis.^5^

No que concerne à pandemia de covid-19, pode-se afirmar que, mesmo antes desse agravante, a população trans já era considerada um grupo vulnerável para IAN,^5,6,7^ porém essa pandemia afetou ainda mais essa realidade.^7,8,9^ Uma das primeiras pesquisas sobre IAN na população trans brasileira identificou uma prevalência de 68,8% na amostra. Além disso, pessoas trans apresentam outros aspectos nutricionais e sociais que são perpassados pela vivência de gênero e há lacunas na literatura científica sobre esse tema, principalmente em países do Sul Global.^7,6^

Com isso, o objetivo deste estudo é descrever a distribuição do estado nutricional e a insegurança alimentar da população trans adulta (acima de 18 anos) da região da Baixada Santista, no estado de São Paulo, e identificar fatores associados. 

## MÉTODOS

### Desenho e população do estudo

Para o presente estudo, foram utilizados dados obtidos na pesquisa Mapeamento da População Trans do Estado de São Paulo: Região Metropolitana da Baixada Santista (MPT), um estudo observacional de corte transversal, com abordagem quantitativa e qualitativa que mapeou a população trans, maior de 18 anos, que residia, trabalhava ou estudava em um dos nove municípios que compõem essa região do estado de São Paulo. O MPT investigou as características socioeconômicas e demográficas dessa população, condições de vida, trabalho, saúde e aspectos relacionados.

### Amostra

Foi aplicado o valor de 1,88% sobre o total da população adulta dos municípios pertencentes à Baixada Santista, única estimativa nacional disponível da magnitude de pessoas trans e não binárias, obtida a partir de uma estimativa brasileira.^10^ Com base em 19.965 pessoas com frequência esperada de 50%, e efeito de desenho de 1, calculou-se uma amostra de 377 pessoas, proporcionalmente distribuída entre os municípios. 

### Recrutamento e coleta de dados

Como estratégia de recrutamento, foi utilizado um cadastramento *on-line* de interesse em participar da pesquisa. O *link* e o código QR de acesso ao cadastro foi amplamente divulgado na região da Baixada Santista e em mídias sociais (Instagram e grupos de WhatsApp da Meta®). Foram convidadas a participar da pesquisa as pessoas trans, travestis e não binárias que preenchessem esse cadastro e que respondessem positivamente à questão *Podemos entrar em contato com você?*, disponibilizando meios de contato para esse fim. 

Para viabilizar o contato com as pessoas que haviam concordado em participar da pesquisa, foi gerada uma planilha em Excel a partir da base presente na plataforma REDCap (Research Electronic Data Capture), a qual era atualizada semanalmente e ficava disponível para a supervisora de campo e para um entrevistador treinado realizar os agendamentos. Esses contatos ocorreram via WhatsApp (Meta®), por *e-mail* ou telefone, e para aquelas pessoas que concordassem em participar era agendada uma entrevista, presencial ou remota, utilizando-se a plataforma Zoom®, a depender da escolha.

Além do cadastro, buscas ativas foram realizadas em unidades básicas de saúde de referência para acompanhamentos em saúde para a população trans, assim como em locais de referência para acolhimento dessa população na região do estudo.

Para a coleta de dados, foi aplicado um questionário estruturado na plataforma REDCap. O questionário completo contava com 11 blocos temáticos (Quadro 1); para este estudo, utilizamos as variáveis dos blocos 1, 2 e 4. 

**Quadro 1:** Blocos temáticos do questionário utilizado na pesquisa Mapeamento da População Trans do estado de São Paulo: Região Metropolitana da Baixada Santista, agosto a dezembro de 2023

Blocos
Características socioeconômicas e demográficas
Acesso a serviços de saúde e rede de apoio
Doenças, problemas de saúde bucal e deficiências
Estado nutricional e acesso à alimentação
Modificações corporais
Comportamento sexual
Saúde sexual, reprodutiva e estratégias de prevenção
Infecções sexualmente transmissíveis (IST)
Qualidade de vida, atividade física e psicossocial
Uso de álcool, tabaco e outras substâncias
Estigma interseccional

O período de coleta de dados para o presente estudo, planejado para outubro e novembro de 2023, foi estendido por mais um mês, com o objetivo de aproximação da amostra inicialmente calculada. Mesmo assim, a amostra não foi alcançada em todos os municípios, e, em razão do esgotamento das estratégias de captação, foi decidida a finalização do campo. 

### Variáveis 

Consideramos como desfechos primários deste estudo o estado nutricional (EN) e a IAN. Para análise das características socioeconômicas e demográficas, foram selecionadas as seguintes variáveis: idade (18 a 24, 25 a 30 e acima de 30 anos); identidades de gênero coletadas como mulher trans, travesti, homem trans, pessoa transmasculina, pessoa não binária, agênero e pangênero e categorizadas em “transfemininas” (mulheres trans e travestis), “transmasculinas” (homem trans e transmasculino) e “não binárias” (pessoa não binária, agênero e pangênero); raça/cor da pele autodeclarada; escolaridade (ensino fundamental incompleto e completo, ensino médio incompleto e completo, ensino superior incompleto e completo, e pós-graduação); renda no último mês (sem renda, até um salário mínimo e acima de um salário mínimo); dificuldade de encontrar um emprego (sim ou não); origem da renda (estágio/estudante bolsista, trabalho formal (Consolidação das Leis do Trabalho – CLT, pessoa jurídica – PJ, servidor público, contrato, trabalho informal e benefício – pensão, Bolsa Família, auxílio-doença e aposentadoria pela previdência social); e existência de apoio familiar, sendo a respondente uma pessoa trans (família não sabe sobre a transgeneridade, apoia completamente, apoia parcialmente, não apoia, indiferente, e não possui contato com a família/reprova completamente). 

O estado nutricional foi analisado utilizando-se o índice de massa corporal (IMC) [peso/altura^2^ (kg/m^2^)], segundo a classificação da Organização Mundial da Saúde (OMS) para a faixa etária de adultos, que categoriza a população em baixo peso grau III (IMC < 16), baixo peso grau II (IMC de 16 a 16,99), baixo peso grau I (IMC de 17 a 18,49), eutrófico (peso ideal para altura) (18,50 a 24,99), sobrepeso (25 a 29,99), obesidade grau I (30,00 a 34,99), obesidade grau II (35,00 a 39,99) e obesidade grau III (acima de 40). Tais categorias foram reagrupadas em baixo peso (IMC < 18,50 kg/m^2^), eutrofia (18,50 a 24,99 kg/m^2^) e sobrepeso (24,99 a 30,00 kg/m^2^) e obesidade (IMC acima de 30,00 kg/m^2^);^11^ a classificação de IMC para pessoas com 60 anos ou mais utilizada é categorizada em baixo peso (IMC < 22,0 kg/m^2^), eutrofia (22,0 a 27,0 kg/m^2^) e sobrepeso/obesidade (IMC acima de 27 kg/m2);^11^ grau de IAN, calculada de acordo com a Escala Brasileira de Insegurança Alimentar ‒ 2014 (oito questões) e classificada como leve (1-3 pontos), moderada (4-5 pontos) e grave (6-8 pontos);^4,5^ e, para avaliar a qualidade de acesso, foi questionado quais tipos de estabelecimentos próximos à residência vendem frutas e verduras.^12^

### Análise estatística

Os resultados foram apresentados de forma descritiva, incluindo frequência, percentual, média e desvio-padrão. Para a análise dos fatores associados aos desfechos, foi aplicado o teste exato de Fisher, considerando-se o valor de p < 0,05 para a tomada de decisão estatística. Os dados foram analisados utilizando-se o *software* estatístico Jamovi 2.3.21.^14^

### Aspectos éticos

O projeto de pesquisa MPT foi submetido e aprovado pelos Comitês de Ética em Pesquisa do Centro de Referência e Treinamento em IST/Aids de São Paulo (CRT) e da Universidade Federal de São Paulo, sob o Certificado de Apresentação de Apreciação Ética nº 64010722.8.0000.5375.

## RESULTADOS

A amostra foi constituída por 237 pessoas, 62,9% da amostra calculada, dos nove municípios da Região Metropolitana da Baixada Santista, sendo os seguintes os municípios mais representados: Santos (27,8%), São Vicente (23,6%), Guarujá (23,2%), Praia Grande (8,9%) e Itanhaém (8,9%). Com relação à identidade de gênero, 42,6% das pessoas se identificaram como transfemininas; 36,3%, como transmasculinas; e 21,1%, como não binárias. A idade mediana encontrada foi de 27 anos (mínima de 18 e máxima de 68 anos), e 67,5% tinham menos de 30 anos.

A Tabela 1 apresenta as características socioeconômicas e demográficas selecionadas. Quanto ao grau de instrução, 80,5% concluíram, no mínimo, o ensino médio. Entretanto, 5,5% da amostra indicou dificuldade para ler e escrever. Em relação à renda e ao mercado de trabalho, 65,8% afirmaram ter dificuldade de encontrar emprego, 63,7% declararam não ter tido nenhuma renda ou renda de até um salário mínimo nos 30 dias que antecederam a entrevista, e, entre as que tinham fonte de renda, 46,3% declararam vínculo de trabalho formal.

O apoio familiar completo (34,9%) e parcial (32,3%) à identidade de gênero foi indicado por 67,2% das pessoas. Cerca de 5% indicaram que a família não sabe sobre sua identidade de gênero trans e 27,7% relataram algum tipo de não apoio familiar. 

As pessoas participantes apresentaram um IMC médio de 25,7 ± 5,57 kg/m^2^ e 50,8% estavam com sobrepeso e obesidade. A IAN estava presente em quase 65% das pessoas da amostra, sendo que, destas, 53,3% foram categorizadas com IAN moderada e grave (Tabela 1).

Com relação ao acesso aos locais que vendem frutas e verduras, próximos à residência da pessoa participante, os mais citados foram mercados de bairro, feira livre, hiper e supermercados de grandes redes e hortifrútis, respectivamente. Relataram não comprar hortifrúti, ou não comprar esses alimentos próximos à residência, 14,8% das pessoas participantes (Tabela 1). 

**Tabela 1:** Características socioeconômicas, demográficas e nutricionais (n e %) de pessoas trans participantes do Mapeamento da População Trans do estado de São Paulo: Região Metropolitana da Baixada Santista, agosto a dezembro de 2023

Variável	Frequência (n)	Percentual (%)
Identidade de gênero (n = 237)		
Transfeminina	101	42,6
Transmasculina	86	36,3
Não binária	50	21,1
**Faixa etária (n = 221)**		
18 a 24 anos	83	37,6
25 a 30 anos	66	29,9
Acima de 30 anos	72	32,6
**Raça/cor da pele (n = 237)**		
Branca	121	51,1
Negra	107	45,1
Outras	9	7,8
**Escolaridade (n = 237)**		
Ensino fundamental incompleto	16	6,8
Ensino fundamental completo	8	3,4
Ensino médio incompleto	22	9,3
Ensino médio completo	92	38,8
Ensino superior incompleto	61	25,7
Ensino superior completo	24	10,1
Pós-graduação	14	5,9
**Renda no último mês (n = 236)**		
Sem renda	77	32,5
Até 1 salário mínimo	74	31,2
Acima de 1 salário mínimo	85	35,9
**Origem da renda (n = 160)**		
Estudante bolsista/estágio	6	3,7
Benefícios sociais e previdenciários	18	11,3
Trabalho informal	62	38,7
Trabalho formal	74	46,3
**Dificuldade para encontrar emprego (n = 237)**		
Sim	156	65,8
Não	73	30,8
Não sabe/não quis responder	8	3,4
**Apoio familiar (n = 235)**		
Apoia completamente	82	34,9
Apoia parcialmente	76	32,3
Não sabe	12	5,1
Indiferente	31	13,2
Não apoia	19	8,1
Reprova completamente	15	6,4
**Estado nutricional (n = 236)**		
Baixo peso	6	2,5
Eutrofia	110	46,6
Sobrepeso	76	32,2
Obesidade	44	18,6
**Insegurança alimentar (n = 237)**		
Sim	150	63,3
Não	87	36,7
**Grau de insegurança alimentar (n = 150)**		
Leve	70	46,7
Moderada	32	21,3
Grave	48	32,0
**Locais que vendem frutas e verduras próximos à residência (n = 237)**		
Supermercado/hipermercado	126	53,2
Mercado de bairro	161	67,9
Sacolão/hortifrúti	107	45,1
Feira livre	155	65,4
Ambulantes	51	21,5
Mercado municipal	10	4,2
Não há estabelecimentos desse tipo	7	3
**Você compra frutas e verduras nos locais próximos à sua residência (n = 237)**		
Sim	202	85,2
Não	29	12,3
Não compro frutas e verduras	6	2,5

Considerando-se a análise bivariada, os fatores associados à presença de IAN foram a dificuldade de encontrar emprego (p < 0,001) (Figura 1) e não possuir nenhuma renda no mês anterior à entrevista (p = 0,009) (Figura 2). Além disso, as pessoas participantes que relataram receber menos de 2 salários mínimos (81%) tinham mais chances de apresentar algum grau de IAN (p < 0,001). Vale ressaltar que não ter renda esteve associado à faixa etária de 18 a 24 anos (p = 0,001).

**Figura 1:** Insegurança alimentar entre população trans segundo a dificuldade de encontrar emprego, conforme dados obtidos pelo Mapeamento da População Trans do estado de São Paulo: Região da Baixada Santista, de agosto a dezembro de 2023, com valores expressos em frequência (n) e percentual (%)

**Figura 2:** Insegurança alimentar da população trans segundo faixas de renda nos últimos 30 dias, conforme dados obtidos pelo Mapeamento da População Trans do estado de São Paulo: Região da Baixada Santista, de agosto a dezembro de 2023, com valores expressos em frequência (n) e percentual (%)

Para além do aspecto de renda e empregabilidade, foi observada associação positiva entre o não apoio familiar e a presença de IAN. As pessoas participantes que relataram apoio parcial ou total por parte da família apresentaram menor probabilidade de terem IAN (p = 0,006); por outro lado, aquelas que relataram indiferença, não apoio ou reprovação da família, por serem pessoas trans, apresentaram mais chance de ter IAN moderada e grave (p = 0,001). Outro aspecto relacionado a esses graus de IAN foi o relato de não comprar frutas e verduras próximo à residência ou da não adquirir hortifrútis rotineiramente (p = 0,050). 

O estado nutricional esteve associado a alguns fatores socioeconômicos. Ler e escrever com dificuldade esteve associado à classificação de sobrepeso e negativamente associado à eutrofia (p = 0,025). Por sua vez, as pessoas com acesso a estabelecimentos que vendem frutas e verduras, como presença de feira livre próxima à residência, apresentaram maior probabilidade de serem classificadas como tendo estado nutricional adequado do que aquelas com sobrepeso e obesidade (p = 0,033) (Figura 3).

**Figura 3:** Classificação do estado nutricional segundo a existência de feira livre próxima à residência, conforme dados obtidos pelo Mapeamento da População Trans do estado de São Paulo: Região da Baixada Santista, de agosto a dezembro de 2023, com valores expressos em frequência (n) e percentual (%)

Nessa amostra, variáveis de raça/cor da pele e identidade de gênero não estavam associadas a nenhuma variável socioeconômica e de insegurança alimentar.

## DISCUSSÃO

O estudo identificou uma alta prevalência de IAN na população estudada, a qual se apresentou associada à renda, à dificuldade de encontrar emprego e à falta de apoio familiar relacionada ao gênero, assim como a aspectos de acesso à aquisição de hortaliças e frutas. 

No Brasil, segundo o relatório da Organização das Nações Unidas para a Alimentação e a Agricultura (FAO), cerca de 34% da população está em situação de IAN.^15^ No entanto, os dados com relação à IAN na população trans no país chegam ao dobro.^8^ Esse estudo, realizado durante a pandemia de covid-19, encontrou prevalência de IAN em uma amostra de 109 pessoas trans brasileiras de todas as regiões do país semelhante ao presente estudo (68,8% *versus* 63,3%). Os autores ressaltam que o acesso à alimentação adequada para essa população já era difícil, mesmo antes da pandemia, e que as principais associações encontradas para essa prevalência se deviam à renda e à falta de emprego,^8^ assim como no presente estudo.

Para se discutir a alta vulnerabilidade social e a IAN na população trans, é necessário levar em consideração a discriminação baseada na identidade de gênero,^8^ assim como as disparidades socioeconômicas entre pessoas trans, que podem estar associadas à passabilidade relacionada ao tempo de transição, à aceitação social e ao suporte familiar.^8^ Provavelmente, a alta proporção de pessoas em IAN do presente estudo, associada à falta de apoio familiar, altas taxas de dificuldade para acessar empregos e, consequentemente, à baixa renda, se relacionam à discriminação baseada na identidade de gênero e transfobia estrutural.^7,8^

Com relação ao estado nutricional, uma revisão sistemática indicou que há uma maior prevalência de sobrepeso e obesidade na população trans, principalmente após o uso de esteroides sexuais para as modificações fenotípicas e corporais.^7^ No presente estudo, não avaliamos o uso de hormônios, porém a prevalência e a distribuição do estado nutricional, segundo as categorias de classificação do IMC, foram semelhantes àquela da população adulta geral do município de São Paulo, apesar de a base de dados considerada ser de 2015, decorrente do Inquérito de Saúde Base Populacional ISA Capital.^16^

Além disso, o estado nutricional eutrófico da amostra estava associado à presença de feiras livres próximas à residência. Um estudo brasileiro indicou que um ambiente com acesso favorável a uma maior variedade de alimentos saudáveis, como frutas e hortaliças, pode favorecer a prevalência de menor sobrepeso e obesidade.^17^

Com relação às limitações do estudo, os dados de peso e altura, utilizados para o cálculo do IMC, foram autorreferidos. Na amostra, a aplicação da mesma porcentagem em todos os municípios pode não corresponder à realidade de distribuição dessa população no território. Além disso, por ser de uma amostra de apenas uma região metropolitana do estado de São Paulo e só ter alcançado 62,9% da amostra calculada, as conclusões deste estudo podem não ser representativas de toda a população trans adulta.

Há poucas pesquisas sobre aspectos nutricionais gerais na população trans, principalmente em países subdesenvolvidos e situados no Sul Global.^6^ Ademais, as pesquisas nacionais produzidas pelo Instituto Brasileiro de Geografia e Estatística (IBGE) não utilizam categorias de gênero trans. Portanto, este estudo contribui com a literatura, dado que há uma lacuna para dados sobre estado nutricional e IAN nessa população, além de existir a possibilidade de comparação com demais pesquisas, por utilizarmos medidas validadas. Portanto, os dados fornecidos por este estudo podem contribuir para discussões embasadas que orientem a necessidade de se delinearem políticas que reduzam a alta prevalência de IAN observada nessa população.

Com isso, recomenda-se que as equipes dos serviços de saúde estejam atentas para a avaliação do estado nutricional e sinais de IAN, em especial profissionais da área de nutrição e assistência social, a fim de se prover um atendimento adequado às demandas e à situação social das pessoas trans atendidas.^6,18^ Os dados desta pesquisa também corroboram a necessidade de políticas públicas e sociais específicas, intersecretariais e intersetoriais, com o objetivo de reduzir as disparidades socioeconômicas aprofundadas pela discriminação,^9^ as quais resultam em uma maior taxa de IAN na população trans. Além disso, faz-se necessária a criação de políticas que visem à facilitação do acesso às feiras livres e demais estabelecimentos que oferecem variedade e qualidade alimentar. 

Essas recomendações estão de acordo com as propostas definidas na 16ª Conferência Nacional de Saúde com relação à capacitação de equipes de saúde para o atendimento de pessoas LGBTI+ e à implementação de políticas públicas de saúde dirigidas para essa população.^19^

Os principais achados deste estudo foram a alta prevalência de IAN entre as pessoas trans, em associação com aquelas mais vulnerabilizadas socialmente e que relataram falta de apoio familiar em função de sua identidade de gênero. Por outro lado, o estado nutricional adequado foi associado à presença de certos estabelecimentos próximos à residência dessa população, como feiras livres. Esses dados indicam a necessidade de políticas públicas e ações voltadas à redução de iniquidades sociais e à ampliação do acesso à alimentação adequada para a população trans.
